# Cellular proteome, coregulators, endocrine system and the human brain: the Regulatory biology of humanism

**DOI:** 10.18632/aging.100270

**Published:** 2011-01-19

**Authors:** Bert W. O'Malley

**Affiliations:** Department of Molecular and Cellular Biology, Baylor College of Medicine, Houston, TX 77030, USA

## The Regulatory Biology of Humanism

There is little doubt that humans are the masters of their known universe. A lively debate could center on why this is so. Although humans have many specialized physical features, such as the marvelous human hand and fingers, other species also have distinguishing features that outclass similar physical features of humans – features such as more acute hearing, smell, or sight. There is little debate, however, that our crowning evolutionary achievement is the human brain. There appears to be no species or other mammal capable of competing with our ability to reason, plan, calculate, and emote. How did this impressive jump in evolution occur? Although not a question that can be answered by hard data, it remains a lively topic for discussion. In my opinion, the two most advanced biologic ‘systems’ that we humans have are (1) the central and peripheral endocrine system and (2) the cellular proteome. It is these two points I would like to further develop in this Perspective.

Our endocrine system and our cellular proteome form an intimate connection with the human brain to construct a powerful troika responsible for our unique capacities as a species. This partnership likely co-evolved to cope with the diversity of our environment and with the intense metabolic demands required to nurture, operate and preserve the human brain. Hormones comprise the endocrine system. The word itself emanates from the Latin word ‘*hormo*’ which means to ‘set in motion’. Hormones do that in great fashion, as they move all cells and organs of our body into action. Our cells and tissues would be relatively inert without them. These chemical regulators control our genesis, development, maturation, and then function in adulthood to support the growth and metabolic functions of all organs, including the brain and its peripheral links. Hormones are not unique to humans, however. They exist even in plants, worms and flies. Mammals, however, have a more highly developed endocrine system that contains many specialized hormones. It is uncertain as to how many hormones actually exist in mammals. We often list ~50 of the more common hormones, but the actual numbers likely are in the hundreds, and perhaps will reach closer to a thousand when finally all are identified. At an earlier period in the field of Endocrinology, we defined hormones as ‘chemical signals released from an organ into the blood stream to act on distant target tissues. Clearly, this definition has been outdated for some years, and a compete list of hormones probably should include a variety of paracrine and autocrine chemical signals, including growth factors, immune cell secretions, cytokines, chemokines, and perhaps even neurotransmitters. There is no greater diversity of environmental signals and physical and emotional stresses for a mammal to bear than those to which a human being has been subjected over the course of evolution.

Although humans have a limited number of unique hormones compared to other mammals, it is our ability to respond in a more complex fashion to our hormones that distinguishes us from other mammals. It is not the number of individual factors but the complexity of combinations that provides our ‘human advantage’. For example, does the total number of keys on a piano provide the complexity to create music, or is it the combinatorial fashion in which the keys are activated by the pianist's fingers. For humans, is the evolutionary advantage we have savored due to the total number of genes we have? Clearly not, as we have virtually the same number of genes as a worm, with current estimates in the neighborhood of ~20,000 each. Consequently, the number of human genes is insufficient to distinguish us from species as distant as fungi or flies. Can the existence of RNA splicing provide sufficient proteomic complexity to distinguish the mammalian species? It appears not. Although humans can splice our genes in five different ways on average, allowing a total of ~125,000 gene products across the genome, worms and flies also can splice their RNAs similarly. How then did mammals achieve the number of combinations of cell signaling events required for the immense complexity that we have achieved? Furthermore, how are we likely to continue to evolve in the very distant future? I believe that the answer to this question lies in our cells' proteomes, and more specifically, in its associated complex signaling pathways.

Perhaps the most defining molecular characteristic of mammals is the greater number of transcriptional coregulators that they possess compared to other species. Our studies in the nuclear receptor field have uncovered a class of new transcriptional regulatory molecules termed coregulators, emanating from our initial cloning of SRC-1 (Steroid Receptor Coactivator-1 [1]. Coregulators are composed of coactivators which provide positive enhancement to gene expression, and corepressors which suppress gene expression. Coactivators are regulatory molecules that are recruited to genes by DNA-binding transcription factors, and provide the ability to fine-tune our genes and activate them in combinations [2]. In this Perspective, we will focus exclusively on our own lab's contributions and on my opinions for the coactivator field, emphasizing the roles of three members of the SRC-family of coactivators.

Recently, we have come to realize that coactivators are the likely ‘master regulators’ of our genome, capable of coordinately activating subgroups of genes that might be required for a specific physiologic process such as growth, reproduction, inflammation, or metabolism [14]. DNA-binding transcription factors, such as nuclear receptors, bind nearby to genes and mark them for activation or repression, functions subsequently effected by the recruitment of coregulators. Worms or flies have few coactivators compared to mammals. Currently, over 350 coregulators have been identified in mammals in the literature (www.NURSA.org]. This number may eventually reach to ~500 or so, given that coactivators and corepressors only have been discovered as a class of molecules for 1.5 decades. We now understand that coactivators act in functional complexes of ~6-12 proteins [3]. This multiplicity of proteins, operating in an active complex, provides great complexity in function due to the large variety of combinations into which different coactivator proteins can assemble in a given coactivator complex [4]. In part, this vast proteomic complexity of transcriptional coactivators allows us a greater genomic complexity for responding to the large variety of environmental signals that impinge upon our cells.

In the proteome, is it all about the primary protein backbones? Absolutely not! The greatest of all defining characteristics of the proteome is the vast number of posttranslational modifications (PTMs) that are superimposed upon our proteins [5]. These include such modifications as phosphorylations, ubiquitinations, sumoylations, methylations, acetylations, glycosyla-tions, etc. We need only to accept the current popular hypothesis that every PTM added to a protein provides it ‘some new capability’ in order to understand the functional power of posttranslational modifications. When a new modification occurs on a protein, we may not be able to understand the precise cellular function of that PTM, but Darwinian logic tells us that if the modification is held constant in the species, then there must be some positive selective advantage for it. The combinatorial potential added to a protein by posttranslational modifications can be enormous. For example, the coactivator, SRC-3, contains over 50 distinct PTMS. This provides an enormous ‘potential’ combinatorial capacity. Consider this theoretical example. If a protein contains 40 PTMs, it has a combinatorial ‘potential’ of (2)^40^, amounting to >1×10^12^ possible combinations [3,6]. Although I am certain that this enormous potential is never reached, my point on the magnitude of potential possibilities is clear; PTMs provide an enormous jump in coactivator protein complexity. Moreover, when we calculate an additional complexity due to the fact that 6-12 proteins work together in combination in a coactivator complex, the numbers reach astronomical proportions. In fact, we probably will never know the true complexity of the mammalian proteome, but it is certainly ‘light years’ beyond what is directly possible from the genome. It is this PTM complexity that imparts the capacity to coactivators to regulate so many diverse cellular reactions, some of which even occur outside the nucleus [3,7].

Although the existence of PTM-controlled coactivators might explain the combinatorial diversity of function required for the superior metabolism and brain power of mammals, one might question why the mouse is not able to function as well as a human when it contains most of the coactivators that have been described in humans. We can only speculate as to the precise explanation, but it appears that humans have more PTMs on their coactivators than do mice. Humans have been reported to have ~4-5 more protein (coactivator) phophorylations than do mice [8]. Although 4 more PTMs may not be overly impressive at first glance, this number provides 16-fold more combinatorial potential per protein, and since each coactivator protein works in a complex with 6-12 other proteins, the factorial calculation of combinations in the complex quickly becomes exponentially large. Also, while it is accepted that rodents can potentially avail themselves to some PTM complexity, their nervous system is not structurally prepared to make full use of PTM complexity as can we humans. It should be remembered that phosphorylation is only one type of PTM modification among many that occur commonly on proteins. There is no doubt that the human brain (and organs) requires large regulatory complexity, and it is most certainly available in the potential of the proteome. One might surmise that in our entire future evolution, we will never have a need for a new gene, since a new function could be acquired simply by adding a novel PTM to an existing protein.

Although humans may owe a part of their evolved capacity as ‘masters of their universe’ to their cadre of ‘master gene’ products, such as the mammalian coregulators, this considerable regulatory advantage comes at a price. Disease processes are prone to co-opt coactivators for their own purposes because they can provide ‘one-stop’ shopping for control of vast numbers of genes that support their pathologic goals. There is no greater example of this than that provided by the cancer cell. A large variety of cancers over-express SRC-3 to promote their relentless growth, including breast, lung, pancreatic, prostate, intestinal and ovarian cancers [9]. Cancers that over-express this coactivator grow more aggressively and are more resistant to therapy. Similarly, SRC-1 has been recently revealed to be a coactivator that promotes tumor metastases [10]. Simultaneous over-expression of both SRC-3 and SRC-2 marks a tumor as particularly dangerous and more likely to recur after therapy.

Metabolism is another major focus of coactivator action. PGC-1 was the first coactivator to be shown by the Spiegelman lab to have metabolic regulatory functions. Recently, we published that the coactivator, SRC-2, is a master gene for energy accretion and storage. When ATP levels in cells drop, SRC-2 is activated by AMP kinase, and SRC-2 then stimulates the liver to secrete bile to the intestine in order to absorb fat from the diet [11]. When the SRC-2 gene is deleted, animals cannot absorb fat efficiently, and cannot store it well. SRC-2 also regulates glucose release from the liver [12]. Thus, this coactivator acts as a sensor for energy accretion, whereby a drop in cellular energy (ATP), leads to an increase in fat (caloric) absorption to replenish whole body energy levels. The SRC-2 coactivator was likely a major advantage for early humans (and mammals) because of their critical need for food, a scarce commodity. Their caloric intake was accomplished best via efficient fat ingestion and storage. In present times, however, with the availability of fast food on most urban corners, SRC-2's efficiency has now become disadvantageous. In any event, these examples serve to substantiate the important roles coactivators play in our metabolic systems biology [13].

Surprisingly, there are very few studies to date on the effects of coactivator dysfunction on the aging process. Observations show that animals with coactivator mutations do not age well, and that early death is more common. Certain alterations in the metabolism with genetically altered coactivators are similar to the aging human. Nevertheless, the lack of experimental studies limits our knowledge of their specific roles. This area of investigation speaks out for more future investigative attention.

In summary, during the past 15 years, we have identified a large new class of regulators, the coactivators/corepressors [14]. A good deal has been learned about their mechanisms of action and their immense physiologic and pathologic relevance. In fact, studies of coactivators may prove key to unlocking the mysteries of polygenic diseases [15]. Now, the challenge arises for us to harness them for therapeutic purposes. Already, they have been demonstrated to have diagnostic and prognostic value for cancers [9]. The question remains as to how we might design a new class of drugs that would regulate these ‘regulators’. Early and mostly unpublished experimental data is encouraging in this respect. Nevertheless, the future will provide a final answer as to whether a new type of pharmacologic intervention at the coactivator level can be utilized for therapies of human diseases.
